# Comparative Analysis of Bacteriophytochrome Agp2 and Its Engineered Photoactivatable NIR Fluorescent Proteins PAiRFP1 and PAiRFP2

**DOI:** 10.3390/biom10091286

**Published:** 2020-09-07

**Authors:** Faez Iqbal Khan, Fakhrul Hassan, Razique Anwer, Feng Juan, Dakun Lai

**Affiliations:** 1 School of Electronic Science and Engineering, University of Electronic Science and Technology of China, Chengdu 610054, China; khanfaeziqbal@gmail.com; 2 School of Life Science and Technology, University of Electronic Science and Technology of China, Chengdu 610054, China; fakhar_ibneadam@yahoo.com (F.H.); fengjuan@uestc.edu.cn (F.J.); 3 Department of Pathology, College of Medicine, Imam Mohammad ibn Saud Islamic University (IMSIU), Riyadh 13317, Saudi Arabia; razainuddin@imamu.edu.sa

**Keywords:** Agp2, PAiRFP1, PAiRFP2, protein folding and stability, molecular dynamics simulation

## Abstract

Two photoactivatable near infrared fluorescent proteins (NIR FPs) named “PAiRFP1” and “PAiRFP2” are formed by directed molecular evolution from Agp2, a bathy bacteriophytochrome of *Agrobacterium tumefaciens* C58. There are 15 and 24 amino acid substitutions in the structure of PAiRFP1 and PAiRFP2, respectively. A comprehensive molecular exploration of these bacteriophytochrome photoreceptors (BphPs) are required to understand the structure dynamics. In this study, the NIR fluorescence emission spectra for PAiRFP1 were recorded upon repeated excitation and the fluorescence intensity of PAiRFP1 tends to increase as the irradiation time was prolonged. We also predicted that mutations Q168L, V244F, and A480V in Agp2 will enhance the molecular stability and flexibility. During molecular dynamics (MD) simulations, the average root mean square deviations of Agp2, PAiRFP1, and PAiRFP2 were found to be 0.40, 0.49, and 0.48 nm, respectively. The structure of PAiRFP1 and PAiRFP2 were more deviated than Agp2 from its native conformation and the hydrophobic regions that were buried in PAiRFP1 and PAiRFP2 core exposed to solvent molecules. The eigenvalues and the trace of covariance matrix were found to be high for PAiRFP1 (597.90 nm^2^) and PAiRFP2 (726.74 nm^2^) when compared with Agp2 (535.79 nm^2^). It was also found that PAiRFP1 has more sharp Gibbs free energy global minima than Agp2 and PAiRFP2. This comparative analysis will help to gain deeper understanding on the structural changes during the evolution of photoactivatable NIR FPs. Further work can be carried out by combining PCR-based directed mutagenesis and spectroscopic methods to provide strategies for the rational designing of these PAiRFPs.

## 1. Introduction

Bacteriophytochrome photoreceptors (BphPs) are red and far-red light sensing proteins predominantly found in certain nonphotosynthetic and photosynthetic bacteria [1,2]. Several BphPs absorb and emit light in near infrared (NIR) region that provide an opportunity for deep tissue in vivo imaging. Mammalian tissues show maximum *trans*parency called as NIR tissues *trans*parency window (650–900 nm). The use of NIR light for in vivo imaging significantly reduces the absorption of light by water, melanin, and hemoglobin (Hb) [3,4]. Previous studies revealed that BphPs exhibit strong absorbance and weak fluorescence in NIR region [5,6]. BphPs have gained much consideration to use them as a template for the development of genetically engineered near infrared fluorescent proteins (NIR FPs) [7,8].

Several engineered canonical BphPs have been successfully developed into permanent FPs. Such types of engineered NIR FPs were mostly developed by truncation of the PHY domain to suppress the photoconversion from *Pr→Pfr* and to increase the NIR fluorescence of *Pr* form [9,10,11,12,13,14,15,16]. Despite much effort, these permanent FPs have low NIR fluorescence quantum yield, therefore resulting in poor signal-to-noise ratio (SNR) for in vivo imaging.

To improve SNR of these NIR fluorescent probes for imaging, Piatkevich et al. successfully screened out two photoactivatable (PA) NIR FPs, named “PAiRFP1” and “PAiRFP2,” by directed molecular evolution from one bathy bacteriophytochrome of *Agrobacterium tumefaciens* C58 (Agp2 or AtBphP2; PDB ID: 6G1Y) [17,18]. *Agrobacterium tumefaciens* C58 is a soil borne pathogen, which causes crown gall disease in plants. It contains one interesting pair of canonical and bathy phytochromes called Agp1 and Agp2. These phytochromes are photochemically different from each other and adopt different thermostable ground states. Agp2 acquires far-red light absorbing (Pfr) as a ground state and generates red light absorbing (Pr) form upon absorption of far-red light. The *Pr* form of Agp2 is unstable and quickly reverts back nonphotochemically to *Pfr* state. When the C-terminal end of Agp2 protein is truncated, then it is called as *Agrobacterium tumefaciens* photosensory core domain (AtPCD). The *Pr* form of AtPCD protein is not stable and experiences quick dark reversion into *Pfr* state with a half time of 11 s [19,20].

To stabilize the *Pr* form and to increase its fluorescence without affecting the *Pfr→Pr* photoconversion, Piatkevich et al. used the AtPCD encoding gene through multiple rounds of directed molecular evolution. In each round, a library of different mutants was constructed by random mutagenesis. After the first round, they identified a weak PA mutant known as At/660-1#1, which contained five substitutions as compared to AtPCD. The nonfluorescent *Pfr* form of this mutant photoconverted into *Pr* state upon absorption at 660 and 750 nm light. After photoconversion, the mutant At/660-I#1 revert back to their initial state with a half-time of 4.1 min. Furthermore, the At/660-I#1 was used as a template for random mutagenesis and after multiple rounds of molecular directed evolution they screened out two different PA FPs, termed as PAiRFP1 and PAiRFP2 [17,19].

Amino acid sequence alignment revealed that PAiRFP1 gained 15 amino acid substitutions during the molecular evolution from Agp2. Twelve out of 15 substitutions are located in GAF (cGMP phosphodiesterase/adenyl cyclase/FhlA cGMP specific phosphodiesterase/adenylate cyclase and (FhIA) formate hydrogen lyase *trans*cription activator) domain while the remaining 3 are positioned in PHY (phytochrome-specific) domain. The PAiRFP2 acquired 24 amino acid substitutions during their molecular evolution from Agp2. Three out of 24 substitutions are located in PAS (“period clock protein” (Per), “aromatic hydrocarbon receptor nuclear *trans*locator” (ARNT), and “single-minded protein” (Sim) domain, 11 in GAF domain, and 10 are in PHY domain [17]. These mutations reduced the dark reversion rates and led to increase in brightness and photoactivation contrast. The fluorescence quantum yield (%) of PAiRFP1 (4.8 ± 0.1) and PAiRFP2 (4.7 ± 0.1) is higher in photoactivated form as compared to AtPCD (0.13). The dark reversion rate of PAiRFP1 (58 min) is fourfold faster than PAiRFP2 (233 min) but displayed higher photoactivation contrast. Different from other BphP-based NIR FPs, these two PA NIR FPs contain PHY domain, which is essential for *Pfr→Pr* photoconversion. Unlike their template Agp2, both PAiRFP1 and PAiRFP2 perform photoactivation behavior because (1) *Pr* and *Pfr* states coexisted in “resting” or “off” state, (2) *Pfr* state can be photoconverted into *Pr* state upon absorbing far-red light, (3) *Pr* state can emit NIR fluorescence, and (4) the *Pr→Pfr* dark recovery becomes slower in PAiRFP1 and PAiRFP2 [18].

Both PAiRFP1 and PAiRFP2 were used for imaging in cultured mammalian Hela cell lines. The fluorescence signal of these PA NIR FPs was equally distributed within the cytosol of living Hela cells without aggregation and nonspecific localization. The PAiRFP1 and PAiRFP2 displayed 25% and 7% effective brightness in cells relative to permanent iRFPs. These PA NIR FPs were also used for in vivo imaging in tumors of living mice, resulting in the higher SNR values and brightness as compared to permanent fluorescent iRFPs [13,17]. Although, these proteins are less bright in mammalian cells as compared to iRFPs, their brightness is enough for imaging of cultured cells and in living mice. The difference in the in vitro brightness and in mammalian cells are due to the lower affinity of biliverdin (BV) and instability of holoprotein. However, imaging with these proteins do not require exogenous supply of BV, as endogenous BV in mammalian cells is sufficient for the formation of fluorescent apoproteins [17]. The photoactivation behavior can change the fluorescence signal of PAiRFPs and allow us to substantially enhance the SNR values for in vivo imaging. The possibility to subtract the PAiRFPs images before and after photoactivation makes these proteins more advantageous over permanent NIR FPs for in vivo imaging when there are typically high autofluorescence environments [4,17].

The present work focuses on studying Agp2, PAiRFP1, and PAiRFP2, to gain a deeper understanding of its structural dynamics. Several computational methods were used to predict the impact of amino acid substitutions on the molecular flexibility and stability of Agp2, PAiRFP1, and PAiRFP2. The 3D structures were modelled and followed by docking of BV in *cis* and *trans* conformations to explore the residual interactions in *Pr* and *Pfr* states of Agp2, PAiRFP1, and PAiRFP2. Several 100 ns MD simulations were performed on three targeted bacteriophytochromes to provide some structural information on the basis of root mean square deviation (RMSD), root mean square fluctuations (RMSF), radius of gyration (Rg), solvent accessible surface area (SASA), average number of hydrogen bonds, secondary structural analysis, principal component analysis, and Gibbs free energy landscapes [21,22,23,24].

## 2. Materials and Methods

### 2.1. Protein Expression, Purification, and In Vitro Assembly with BV

The *Escherichia coli* MC1061 cells were used as a host cell for the expression of PAiRFP1 protein as described in ref [18] with some modifications. The proteins were purified with nickel-affinity His-trap chelating column equipped with an AKTA purifier system (GE Healthcare). The resulting apoproteins were assembled at ~3× molar excess of BV for overnight at 4 °C to allow holoprotein formation [25]. NAP-10 desalting column (GE Healthcare) was used to remove the unbound BV. The proteins were stored in a 50 mM Tris-HCl buffer at pH 7.8 for further use.

### 2.2. Spectroscopic Measurements

The NIR fluorescence emission spectra were detected upon repeated excitation at 680 nm for 10 min, using the F4600 spectrofluorometer (Hitachi). The protein samples were kept in dark and measured before and after repetitive irradiation using a 680 nm incident beam for 10 min. The fluorescence intensity and time-dependent dynamic curve for PAiRFP1 was plotted at 1–10 min immediately after repeated excitation. The UV–VIS NIR absorption spectra were recorded using U2910 spectrophotometer (Hitachi) at room temperature.

### 2.3. 3D Structure Modelling

The three-dimensional (3D) structure of these BphPs was generated by homology modelling method using Modeller 9.10 [26,27]. The structural homologue search was performed in the Protein Data Bank (PDB) using basic local alignment search tool [28]. The missing residues in the crystal structures of Agp2 and PAiRFP2 were also modelled in order to obtain proper structure of Agp2, PAiRFP1, and PAiRFP2. The sequence of PAiRFP1 and PAiRFP2 were aligned with their templates Agp2 (PDB ID: 6G1Y) and PAiRFP2 (PDB ID: 6G1Z). The fold recognition methods were used to optimize the sequence-structure alignment. The most reliable models were evaluated on the basis of RMSD, TM score, and DOPE profile. The selected models were further refined using SCWRL 4.0 [29], and the GROMOS 43B1 force field implemented in Swiss-PdbViewer was used for energy minimization of the predicted 3D structures [30].

### 2.4. Mutation Analysis

Several computational servers such as DynaMut, DeepDDG, and Discovery Studio were used to predict the impact of mutations on the molecular flexibility and stability of proteins [31,32]. DynaMut is a powerful computational tool, which can be used to analyze the effect of single-point mutation on the stability and dynamics of protein resulting from vibrational entropy changes. It operates through Normal Mode Analysis (NMA) by implementing two different approaches Bio3D and ENCoM, which provide rapid, simplified, and powerful insightful exploration of protein dynamics [33,34,35,36], whereas DeepDDG server predicts the stability change of protein point mutations using neural networks [37]. These predictors were successfully applied to all the mutated sites in PAiRFP1 and PAiRFP2 to compute the impact of single-point mutations on the basis of protein folding free energy (ΔΔG) and vibrational entropy changes (ΔΔS) [31,38,39,40,41].

### 2.5. Docking Studies

The literature review suggested that BV in BphPs tends to be in *cis* and *trans* form in *Pr* and *Pfr* states, respectively [42,43,44]. In order to understand the phenomena of *Pr* and *Pfr* conformations, the molecular docking of BV in *cis* and *trans* forms were performed with Agp2, PAiRFP1, and PAiRFP2 by Schrodinger molecular modelling software using CovDock that performs a series of automated steps based on a simple setup from the Maestro graphical interface [45,46]. The best docked poses of BV in both states were selected based on binding energy and proper orientations [25]. We assumed that the docked complex Agp2-BV*cis*, PAiRFP1-BV*cis*, and PAiRFP2-BV*cis* are in *Pr* states, whereas Agp2-BV*trans*, PAiRFP1-BV*trans*, and PAiRFP2-BV*trans* are in *Pfr* states [18].

### 2.6. MD Simulations

Many marvelous biological functions in proteins and their profound dynamic mechanisms can be revealed by studying their internal motions using MD simulation [47,48,49,50,51]. The MD simulations were performed on apoproteins of Agp2, PAiRFP1, and PAiRFP2 at 300 K at the molecular mechanics level implemented in the GROMACS 2018.2 [52] using the GROMOS96 43a1 force field. The Agp2, PAiRFP1, and PAiRFP2 molecules were soaked in a cubic box of water molecules with a dimension of 10 Å, i.e., setting the box edge 10 Å from the molecule periphery using the *gmx editconf* module for creating boundary conditions and *gmx solvate* module for solvation. The simple point charge (spc216) water model was used to solvate the protein. The charges on Agp2, PAiRFP1, and PAiRFP2 molecules were neutralized by addition of Na^+^ and Cl^−^ ions using *gmx genion* module to maintain neutrality, preserving a physiological concentration (0.15 M). The detailed methodology used to analyze the trajectories has been described in our previous communications [51,53,54,55]. All graphical presentations of the 3D models were prepared using PyMOL and VMD (Visual Molecular Dynamics) [56].

### 2.7. Essential Dynamics

The principal component analysis (PCA) or essential dynamics (ED) reflects the overall expansion of a protein during MD simulations. The sum of the eigenvalues is a measure of the overall mobility in the system to relate the elasticity of a protein under different environments. PCA or ED were calculated for atomic motions in Agp2, PAiRFP1, and PAiRFP2 molecules. The PCA is based on the diagonalization of the covariance matrix C, with the elements explained as follows:

C_ij_ = 〈 (r_i_ − 〈r_i_〉) × (r_j_ − 〈r_j_〉) 〉 (i, j = 1,2,3,…,3N) (1)

where r_i_ represents the cartesian coordinate of the i^th^ Cα atom, N is the number of Cα atoms, and <r_i_> indicates the time average over all configurations achieved during MD simulation. The MD projections of trajectories onto the key essential dynamics relates to the largest eigenvector, and the major fluctuations of the correlated atomic motions can be visualized [57].

### 2.8. Gibbs Free Energy Landscape

The structural features and conformational profiles of a protein can be obtained by Gibbs free energy landscape using conformational sampling methods [55]. The structural information and conformations profiles attained by MD simulations are used for further analysis. In order to obtain 2D and 3D depiction, the Gibbs free energy landscapes were projected onto the first principal component (PC1) and second principal component (PC2) with the highest eigenvalues calculated from PCA analysis for Agp2, PAiRFP1, and PAiRFP2. The free energy landscapes are defined by following equation as:

*G*_(PC1, PC2)_ = −*kBT* ln *P*_(PC1, PC2)_ (2)

where *kB* is the Boltzmann constant, T is the temperature, and P_(PC1, PC2)_ is the normalized joint probability distribution.

## 3. Results

### 3.1. Stability of NIR Fluorescence Emission

**Figure 1:**
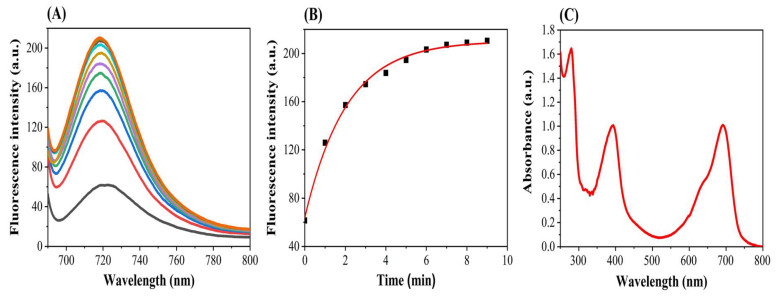
(**A**) NIR fluorescence emission spectra, (**B**) single exponential time-dependent dynamic curve of fluorescence intensity, and (**C**) UV–VIS NIR absorption spectra of PAiRFP1, in 50 mM Tris-HCl buffer (pH 7.8 containing 5 mM EDTA and 300 mM NaCl) after repeated excitation at 680 nm for 10 min.

It is known that BV exhibited strong NIR absorption overlapping with its NIR fluorescence. The protein sample of PAiRFP1 was diluted so as to avoid the self-absorption from *Pr* state and inner filter effect from *Pfr* state. The protein samples were diluted to OD700 of 0.05, and the NIR fluorescence emission spectra were recorded at room temperature upon repeated excitation at 680 nm for 10 min. The single-exponential growth function was used to fit the time-dependent curve of the fluorescence intensity for PAiRFP1 at 1–10 min after repeated excitation. The results showed that the NIR fluorescence intensity tended to increase for PAiRFP1 as the irradiation time was prolonged (Figure 1A,B). This phenomenon could be explained because *Pfr* state photoconverted into *Pr* state upon excitation at 680 nm was observed from the absorption spectra (Figure 1C). Since only *Pr* state can emit NIR fluorescence, it was evident that NIR fluorescence intensity will be enhanced. Different from other BphP-based NIR FPs, PAiRFP1 and PAiRFP2 have a PHY domain, which is essential for *Pfr→Pr* photoconversion [13,17,19,20].

### 3.2. Structure Analysis

The sequence of PAiRFP1 and PAiRFP2 showed 96% and 94% similarity with wild-type Agp2. The obtained structures of the PAiRFP1 and PAiRFP2 exhibited low violations of restraints, and hence it is assumed to be more precise. The final alignment of 3D models of PAiRFP1 and PAiRFP2 with Agp2 displayed a RMSD values of 0.13 and 0.19 Å, respectively. The alignment of PAiRFP1 with PAiRFP2 showed RMSD values of 0.11. The lower RMSD values suggested a higher resemblance of the predicted model with its templates. The PAiRFP1 molecule has G127D, S141R, M163L, Q168L, A203V, G218S, R220P, V244F, A276V, Y280C, E294V, H303R, A386V, A480V, and H498Y mutations, whereas PAiRFP2 has K69R, R83K, G120D, A123T, M163L, Q168E, R220P, S243N, V244F, G269D, A276V, Y280C, E294A, H303F, H333R, I336L, D349R, M351I, A386V, G409D, L419I, T469S, A487T, and E494G mutations. Furthermore, the Verify_3D shows that PAiRFP1 has 88.80% and PAiRFP2 has 93.32% of the entire residues with an averaged 3D-1D score of >0.2. It indicates the reliability and stability of model prediction. The overall quality factor score predicted by ERRAT was found to be 84.63 and 82.24 for PAiRFP1 and PAiRFP2, respectively. The topology for Agp2, PAiRFP1, and PAiRFP2 molecules were obtained by PDBsum to understand the detailed structural qualities [21]. Agp2 has 3 β-sheets, 9 β-hairpins, 9 β-bulges, 18 strands, 20 α-helices, 15 helix-helix interactions, 41 β-turns, and 1 γ turn in its structure. PAiRFP1 has 3 β-sheets, 9 β-hairpins, 10 β-bulges, 18 strands, 18 α-helices, 14 helix-helix interactions, 45 β-turns, and 3 γ turns in its structure, whereas PAiRFP2 has 3 β-sheets, 9 β-hairpins, 10 β-bulges, 18 strands, 20 α-helices, 15 helix-helix interactions, 42 β-turns, and 1 γ turn in its structure. Ramachandran plot of Agp2 showed 93.5% of the entire amino acid residues in the most favored regions, 5.6% in the additional allowed regions, 0.7% in generously allowed regions, and 0.2% in disallowed regions. Ramachandran plot of PAiRFP1 indicated that it has 93.5% of entire residues in most favored regions, 5.8% in additional allowed regions, 0.4% in generously allowed regions, and 0.2% in disallowed regions. Ramachandran plot of PAiRFP2 indicated that it has 93.5% of entire residues in most favored regions, 5.8% in additional allowed regions, 0.4% in generously allowed regions, and 0.2% in disallowed regions (Figure S1).

### 3.3. Hydrogen Bonds and van der Waals Interactions

The Discovery Studio was used to analyze the different kinds of noncovalent interactions at the mutated sites in the predicted models of PAiRFP1 and PAiRFP2 and compared them with Agp2. The H-bonding and van der Waals forces pattern in the predicted protein structures of PAiRFP1 and PAiRFP2 were analyzed by Discovery Studio at the mutated sites, and compared them with Agp2 (Table 1, Table 2 and Table 3). In the PAS domain, mutation at residue K69 and R83 on Agp2 will not affect hydrogen bonding and van der Waals interactions in PAiRFP2. While mutation in G120 of Agp2 will result in loss and gain of van der Waals atomic interactions in PAiRFP2 with residues S121 and S286, respectively. In case of PAiRFP1, the mutation of M163, Q168, and Y280 sites from Agp2 to 163L, 168L, and 280C led to decrease in hydrogen bonding. While, the mutation of other sites from Agp2 did not affect hydrogen bonding. The overall van der Waals forces increased when Q168, Y280, E294, H303, and A480 sites from Agp2 were mutated to 168L, 280C, 294V, 303R, and V480, whereas the mutation of R220 and H498 sites from Agp2 to 220P and 498Y led to decrease in van der Waals forces. In summary, mutations in residues E294, H303, and A480 might improve the stability of Agp2, while residues R220 and H498 have no positive effects on the structural stability of Agp2. Our previous communications also suggested that reverse mutations V480A in PAiRFP1 will decrease the extinction coefficient and relative fluorescence quantum yield [18]. In case of PAiRFP2, the mutation of G120, S243, and E494 sites from Agp2 to 120D, 243N, and 494G led to increase in hydrogen bonding such as D120-S121, N243-Y23-F244, and G494-H498, whereas the mutation of M163, Y280, A487 sites from Agp2 to 163L, 280C, and 487T led to decrease in hydrogen bonding. Additionally, it is found that the overall van der Waals forces increase when Q168, V244, Y280, H333, M351, G409, and A487 sites from Agp2 were mutated to 168E, 244F, 280C, 333R, 351I, 409D, and 487T, whereas the mutation of M163, R220, D349, L419, T469 sites from Agp2 to 163L, 220P, 349R, 419I, and 469S led to decrease in van der Waals forces.

### 3.4. Stability and Flexibility Analysis

The DynaMut and DeepDDG servers were used to predict the impact of mutations on the molecular flexibility and stability of PAiRFP1 and PAiRFP2. These predictors were successfully applied to all the mutated sites in PAiRFP1 and PAiRFP2 to compute the impact of single-point mutation on the basis of protein folding free energy (ΔΔG) and vibrational entropy changes (ΔΔS).

In case of PAiRFP1, the DynaMut suggested that the mutation of G127, Q168, A203, V244, A276, A386, and A480 sites from Agp2 to 127D, 168L, 203V, 244F, 276V, 386V, and 480V led to increase in molecular flexibility and stability of protein, whereas the mutation of S141, M163, G218, R220, Y280, E294, H303 sites from Agp2 to 141R, 163L, 218S, 220P, 280C, 294V, 303R led to decrease in molecular flexibility and stability of protein. Additionally, the DeepDDG server recommended that the mutation of G127, Q168, R220, A386, and A480 sites from Agp2 to 127D, 168L, 220P, 386V, and 480V resulted in an increase in stability of protein, whereas the mutation of S141, M163, A203, G218, V244, A276, Y280, E294, H303, and H498 sites from Agp2 to 141R, 163L, 203V, 218S, 244F, 276V, 280C, 294V, 303R, and 498Y led to decrease in stability of protein (Table 4). The combined results of DynaMut and DeepDDG servers suggested that the mutations G127D, Q168L, A386V, and A480V are important for structural stability and flexibility of PAiRFP1.

In case of PAiRFP2, the DynaMut proposed that the mutation of G120, A123, Q168, S243, V244, A276, H303, I336, D349, A386, G409, L419, and A487 sites from Agp2 to 120D, 123T, 168E, 243N, 244F, 276V, 303F, 336L, 349R, 386V, 409D, 419I, and 487T led to increase in molecular flexibility and stability of protein, whereas the mutation of K69, R83, M163, R220, G269, Y280, E294, H333, M351, T469, and E494 sites from Agp2 to 69R, 83K, 163L, 220P, 269D, 280C, 294A, 333R, 351I, 469S, and 494G led to decrease in molecular flexibility and stability. Further, the DeepDDG recommended that the mutation of K69, G120, R220, H303, I336, M351, A386, and G409 from Agp2 to 69R, 120D, 220P, 303F, 336L, 351I, 386V, and 409D resulting an increase in stability of protein, whereas the mutation of R83, A123, M163, Q168, S243, V244, G269, A276, Y280, E294, H333, D349, L419, T469, A487, and E494 sites from Agp2 to 123T, 163L, 168E, 243N, 244F, 269D, 276V, 280C, 294A, 333R, 349R, 419I, 469S, 487T, and 494G led to decrease in stability of protein (Table 5). The combined results of DynaMut and DeepDDG servers suggested that the mutations G120D, H303F, I336L, A386V, and G409D are important for structural stability and flexibility of PAiRFP2.

In summary, the combined results of hydrogen bonding, van der Waals interactions, and DynaMut and DeepDDG servers predicted that mutations Q168L, V244F, and A480V of Agp2 will enhance the molecular stability and flexibility.

### 3.5. Interaction of BV in Cis and Trans Forms

**Figure 2:**
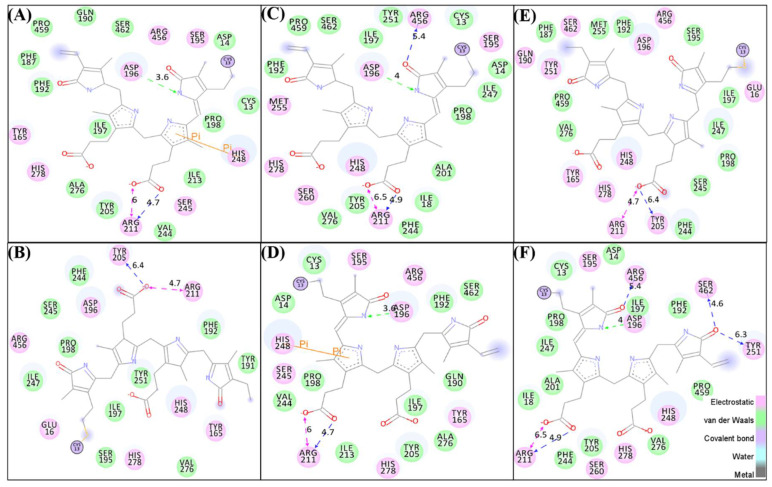
The molecular docking of biliverdin into the active pocket of BphPs. The structure indicated different residual interactions in (**A**) Agp2-BV*cis*, (**B**) Agp2-BV*trans*, (**C**) PAiRFP1-BV*cis*, (**D**) PAiRFP1-BV*trans*, (**E**) PAiRFP2-BV*cis*, and (**F**) PAiRFP2-BV*trans*, respectively.

The BV in *cis* and *trans* forms were docked into the active site of Agp2, PAiRFP1, and PAiRFP2. The top poses of BV in both forms were selected. BV was covalently bound to Cys13 of Agp2, PAiRFP1, and PAiRFP2 as it is present in nature. The superimposition of the docked complexes suggested that BV is bound to Agp2, PAiRFP1, and PAiRFP2 in the same position and proper orientations. All the docked complexes such as Agp2-BV*cis*, Agp2-BV*trans*, PAiRFP1-BV*cis*, PAiRFP1-BV*trans*, PAiRFP2-BV*cis*, and PAiRFP2-BV*trans* were analyzed (Figure 2A–F). These *cis* and *trans* orientations of BV are considered as *Pr* and *Pfr* states of each BphP, respectively. It clearly indicates that the residual interactions change upon different orientations of BV in BphPs (Table 6). The overall van der Waals interactions were increased in case of Agp2-BV*cis* and PAiRFP1-BV*cis* forms as compared to their *trans* orientations of BV. The electrostatic interactions were increased in case of PAiRFP2-BV*cis* form as compared to PAiRFP2-BV*trans*. The van der Waals interactions were also increased in case of PAiRFP2-BV*trans* as compared to PAiRFP2-BV*cis*.

The hydrophobic and hydrophilic SASA for Agp2 were found to be 112.04 and 132.54 nm^2^, respectively. The hydrophobic and hydrophilic SASA for PAiRFP1 were found to be 120.47 and 128.91 nm^2^, respectively. The hydrophobic and hydrophilic SASA for PAiRFP2 were found to be 117.45 and 130.89 nm^2^, respectively. The hydrophobic SASA was increased in case of PAiRFP1 and PAiRFP2 as compared to Agp2. The hydrophilic SASA was decreased in case of PAiRFP1 and PAiRFP2 as compared to Agp2. The hydrophobic SASA was found to increase in case of PAiRFP1 than PAiRFP2. There was a slight decrease in hydrophilic SASA in case of PAiRFP1 than PAiRFP2. The graph clearly shows that the hydrophobic regions that were buried in the PAiRFP1 core were exposed to solvent than PAiRFP2.

### 3.6. Structural Dynamics

#### 3.6.1. Structural Deviations and Compactness

**Figure 3:**
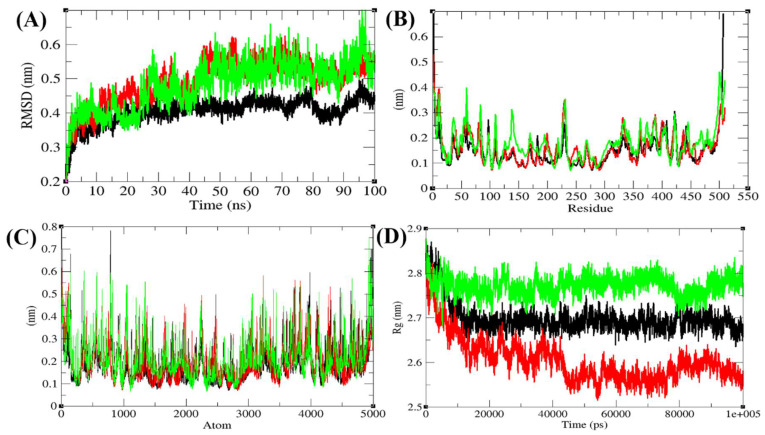
Structural dynamics of Agp2, PAiRFP1, and PAiRFP2. (**A**) Root mean square deviations plot as a function of time. (**B**) Root mean square fluctuations plot vs. residues. (**C**) Root mean square fluctuations plot vs. atoms. (**D**) Time evolution of radius of gyration (*R*_g_). Black, red, and green color represent values obtained for Agp2, PAiRFP1, and PAiRFP2 during 100 ns molecular dynamics (MD) simulations, respectively.

To explore the structural dynamics of Agp2, PAiRFP1, and PAiRFP2, the root mean square deviation (RMSD), root mean square fluctuations, and the radius of gyration (Rg) were analyzed [22]. The average RMSD values of Agp2, PAiRFP1, and PAiRFP2 were found to be 0.40, 0.49, and 0.48 nm, respectively (Figure 3A). Agp2 showed least RMSD fluctuation as compared to PAiRFP1 and PAiRFP2, whereas PAiRFP1 and PAiRFP2 showed similar patterns in the RMSD plot. Further, the root mean square fluctuation (RMSF) of the Agp2, PAiRFP1, and PAiRFP2 were plotted (Figure 3B,C).

The average RMSF of the Agp2, PAiRFP1, and PAiRFP2 were found to be 0.15, 0.16, and 0.18 nm, respectively. The rise in residual fluctuations are due to mutations in the structure of PAiRFP1 and PAiRFP2. The Rg is linked to the tertiary structural volume of Agp2, PAiRFP1, and PAiRFP2. A protein, which has higher Rg is assumed to have less tight packing. The average Rg values for Agp2, PAiRFP1, and PAiRFP2 were found to be 2.70, 2.60, and 2.77 nm, respectively. It is found that PAiRFP1 has more tight packing than Agp2 and PAiRFP2 in its tertiary structure (Figure 3D).

#### 3.6.2. Solvent Accessible Surface Area

**Figure 4:**
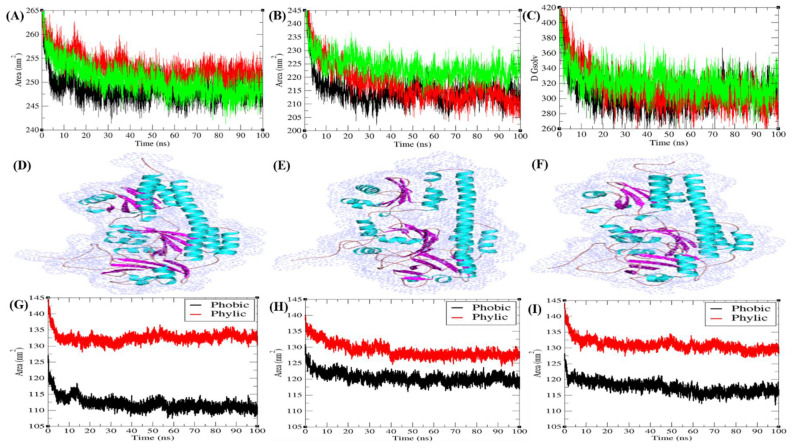
Solvent accessible surface area (SASA). The SASA as a function of time obtained for Agp2, PAiRFP1, and PAiRFP2 with reference to (**A**) backbone and (**B**) protein. (**C**) The free energy of solvation with reference to time. Black, red, and green color represent values obtained for Agp2, PAiRFP1, and PAiRFP2, respectively. The SASA were shown in 3D structure of (**D**) Agp2, (**E**) PAiRFP1, and (**F**) PAiRFP2, respectively. The SASA plots were further resolved into hydrophobic (black) and hydrophilic (red) regions for (**G**) Agp2, (**H**) PAiRFP1, and (**I**) PAiRFP2, respectively.

The solvent accessible surface area (SASA) is explained as the surface area of a protein which forms networks with its solvent molecules [23]. The average SASA values with respect to backbone for Agp2, PAiRFP1, and PAiRFP2 were found to be 248.60, 252.88, and 250.42 nm^2^, respectively (Figure 4A). The average SASA values with respect to protein for Agp2, PAiRFP1, and PAiRFP2 were found to be 215.09, 217.67, and 223.87 nm^2^, respectively (Figure 4B). The SASA plot suggested that PAiRFP1 and PAiRFP2 have more SASA than Agp2 due to point mutations. This can be presumed as the internal residues of PAiRFP1 and PAiRFP2 were exposed to solvent due to these point mutations. Solvation plays crucial roles in monitoring the stability of protein structure. The solvation effect is measured by the solvation free energy and reflects the atomic-level interactions between the protein and solvent. The free energy of solvation for Agp2, PAiRFP1, and PAiRFP2 were found to be 307.15, 313.21, and 321.49 kJ/mol/nm^2^, respectively (Figure 4C). The mutations in Agp2 result in high solvation energy. Additionally, the SASA plots were further resolved into hydrophobic and hydrophilic regions (Figure 4G–I).

#### 3.6.3. Hydrogen Bonds and Secondary Structure Analysis

**Figure 5:**
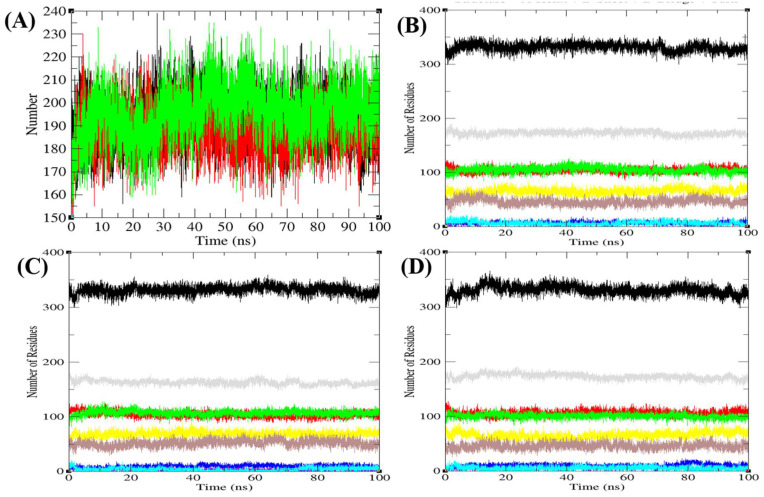
The average number of hydrogen bonds and secondary structure plot as a function of time. (**A**) The average number of hydrogen bonds formation calculated during 100 ns MD simulations between main and side chains of Agp2 (black), PAiRFP1 (red), and PAiRFP2 (green), respectively. The graphical representation indicating structural elements present in (**B**) Agp2, (**C**) PAiRFP1, and (**D**) PAiRFP2 during 100 ns MD simulations. The secondary structure elements represent α-helices, β-sheets, β-bridge, and turns. Each structural element has been represented by different color such as black (overall structure), red (coil), green (β-sheet), blue (β-bridge), yellow (bend), brown (turn), grey (α-helix), and cyan (3-helix), respectively.

The free energy difference (ΔG) between the folded and unfolded states of proteins is affected by changes in atomic interactions. Changes in the interaction among residues within a protein and its surroundings affect the entropy of the system, thus affecting flexibility/rigidity of the structure. Proteins are stabilized by covalent disulfide bonds and the noncovalent hydrophobic, electrostatic, van der Waals, and hydrogen bonds. The hydrogen bond is a significant factor in stabilizing protein conformations. To check the hydrogen bond formations between main and side chains of Agp2, PAiRFP1, and PAiRFP2, the hydrogen bonds paired within 0.35 nm were estimated during the 100 ns MD simulations. The average number of hydrogen bonds between main and side chains of Agp2, PAiRFP1, and PAiRFP2 were found to be 192, 188, and 196, respectively (Figure 5A). The average number of hydrogen bonds decrease in case of PAiRFP1 and increase in case of PAiRFP2. This may be due to the increase in SASA of PAiRFP1.

**Figure 6:**
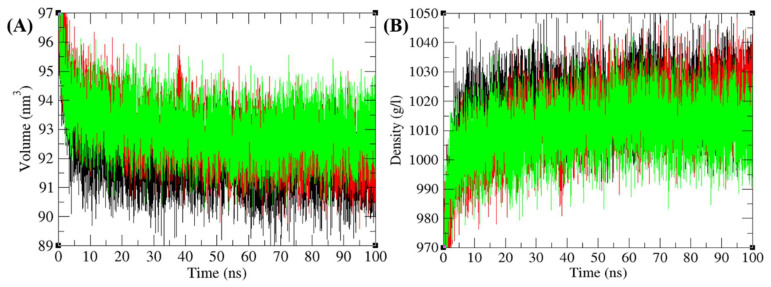
The structural volume and density plot as a function of time. (**A**) The structural volume and (**B**) the density of Agp2 (black), PAiRFP1 (red), and PAiRFP2 (green) calculated during 100 ns MD simulations, respectively.

The purpose of the secondary structure is to spot the structural features of Agp2, PAiRFP1, and PAiRFP2. The secondary structure assignments in Agp2, PAiRFP1, and PAiRFP2 such as α-helix, β-sheet, and turn were split into individual residues at each time step. The average number of residues participated in secondary structure formation of Agp2, PAiRFP1, and PAiRFP2 were compared (Table 7). It was found that the overall average residues participated in structure formation in case of Agp2, PAiRFP1, and PAiRFP2 were the same. In case of PAiRFP1, the α-helix slightly decreases, whereas β-sheets remain the same when compared with Agp2. In case of PAiRFP2, β-sheets slightly decrease, whereas α-helix remains the same (Figure 5B–D). Further, the volume and density of Agp2, PAiRFP1, and PAiRFP2 were calculated. It was found that the volume of Agp2, PAiRFP1, and PAiRFP2 were 91.94, 92.85, and 93.09 nm^3^, respectively. The average density of Agp2, PAiRFP1, and PAiRFP2 were found to be 1018.35, 1013.08, and 1009.72 g/L, respectively (Figure 6A,B). It is clear that the volume of PAiRFP1 and PAiRFP2 are slightly higher than Agp2, which is in line with SASA data. Similarly, the density of PAiRFP1 and PAiRFP2 are less than Agp2.

### 3.7. Principal Component Analysis

**Figure 7:**
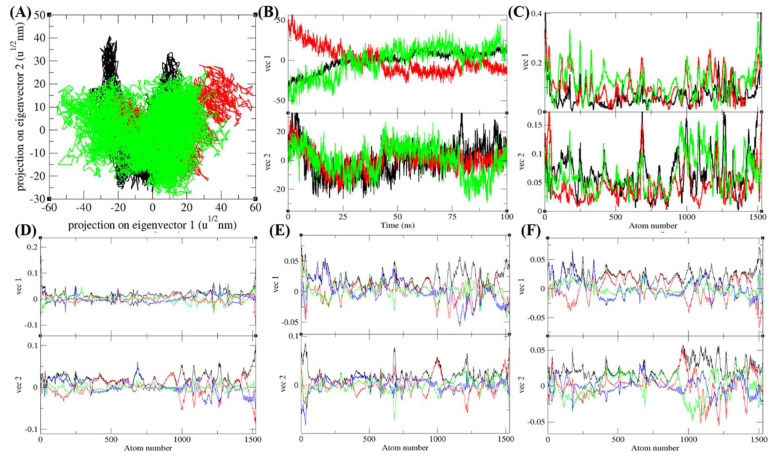
Projection of eigenvectors and components. (**A**) The 2D projections of trajectories on eigenvectors showed different projections of Agp2, PAiRFP1, and PAiRFP2. (**B**) The projections of trajectories on eigenvectors with respect to time. (**C**) Root mean square fluctuations (RMSF) obtained during principal component analysis (PCA) calculations. Black, red, and green color represent the values obtained for Agp2, PAiRFP1, and PAiRFP2, respectively. The eigenvector components were further resolved into total (black), × (red), y (green), and z (blue) direction for (**D**) Agp2, (**E**) PAiRFP1, and (**F**) PAiRFP2, respectively. The graph represents the large-scale average motion in Agp2, PAiRFP1, and PAiRFP2, thus revealed the structures underlying the atomic fluctuations.

The PCA or ED show an overall expansion of Agp2, PAiRFP1, and PAiRFP2 molecules during MD simulation. The PCA for Agp2, PAiRFP1, and PAiRFP2 were calculated using *gmx covar* module with respect to the backbone. The ED identifies the substantial average atomic motions of Agp2, PAiRFP1, and PAiRFP2. The sum of the eigenvalues is a measure of the overall mobility in the system, and it relates with the elasticity of a protein. The eigenvalues and the trace of the covariance matrix were found to be 535.79, 597.90, and 726.74 nm^2^ for Agp2, PAiRFP1, and PAiRFP2, respectively. The eigenvalues and the trace of covariance matrix were found to be high for PAiRFP1 and PAiRFP2 when compared with Agp2. The higher values of eigenvalues and the trace of covariance matrix suggest that the average random fluctuations are more in case of PAiRFP2 than PAiRFP1. Greater eigenvalues indicate more expansion of PAiRFP2 than PAiRFP1. Figure 7A–F shows detailed multidimensional covariance matrix for each atom pair covariance. The 2D projections of trajectories on eigenvectors displayed diverse projections of Agp2, PAiRFP1, and PAiRFP2 molecules. There was a major difference of projections of trajectories in case of PAiRFP2. The variations in the position of atoms are very different in case of PAiRFP2 than PAiRFP1 and Agp2. This might be due to different conformations of PAiRFP2 during MD simulations. The root mean square atomic fluctuations of Agp2, PAiRFP1, and PAiRFP2 were also monitored during the PCA calculations. The random atomic fluctuations of PAiRFP2 were more than PAiRFP1 and Agp2. The eigenvector components were further resolved into x, y, and z directions.

### 3.8. Gibbs Free Energy Landscape

**Figure 8:**
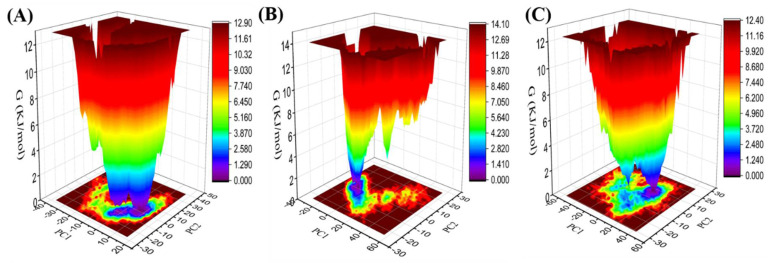
**Gibbs energy landscape.** The Gibbs energy landscape plot obtained during 100 ns MD simulations for (**A**) Agp2, (**B**) PAiRFP1, and (**C**) PAiRFP2, respectively.

The *gmx covar*, *gmx anaeig*, and *gmx sham* modules were utilized to calculate the Gibbs free energy landscape by their own initial (PC1) and next (PC2) eigenvectors projections. The color-coded energy landscape displayed varied forms for Agp2, PAiRFP1, and PAiRFP2 (Figure 8A–C). The covariance matrix for each atom pair covariance shows different patterns in each case. The corresponding free energy contour map with deep blue shade represents lower energy state. The main free energy well in the global free energy minimum region of Agp2, PAiRFP1, and PAiRFP2 is different. A comparison between the full views of the Gibbs free energy values of Agp2, PAiRFP1, and PAiRFP2 suggested that these BphPs have different patterns of global minima. PAiRFP1 has a sharper stable global minimum than Agp2 and PAiRFP2. A single deep blue color well is generally treated as stable. The single sharp stable global minima might be the reason for improved photophysical properties of PAiRFP1.

## 4. Discussion

BphPs contain heme breakdown product, linear tetrapyrrole biliverdin (BV) IXα, as a chromophore. BV is the most abundant and ubiquitous product in cells of different eukaryotic organisms. Most of the BphPs have been successfully used as a template for the development of NIR FPs [5,6]. Low light scattering and reduced autofluorescence of NIR light in biological tissues provide higher SNR for deep tissue imaging as compared to visible light [3,4]. The development of such NIR FPs enable scientists to thoroughly monitor the different biological processes like cellular pH, metabolite concentrations and homeostasis, protein–protein interactions, expression, localization, dynamics, and solubility of proteins [58,59,60,61,62].

In this study, the NIR fluorescence emission spectra for PAiRFP1 were detected upon repeated excitation at 680 nm for 10 min, and it was found that the fluorescence intensity of PAiRFP1 enhanced as the irradiation time was prolonged. The *Pfr* state photoconverted into *Pr* state upon excitation at 680 nm observed from the absorption spectra (Figure 1C). Since only *Pr* state can emit NIR fluorescence, it was evident that NIR fluorescence intensity will be enhanced. Upon absorption of light, BphPs are reversibly photoconverted between two spectrally distinct and relatively stable forms termed as *Pr* and *Pfr* states. The irradiation of *Pr* and *Pfr* forms with red and far-red light lead to *cis* (Z or Zusammen) and *trans* (E or Entgegen) isomerization of C15 = C16 double bond between ring C and D within the bilin chromophore system. This causes a cascade of conformational changes within the protein structure [63]. All the nitrogen atoms of BV become protonated during the stable forms of *Pr* and *Pfr* states and briefly deprotonated at ring B or C during *Pr→Pfr* photoconversion [64]. The canonical BphPs gain stable *Pr* state in dark and produce *Pfr* state upon illumination with red light. The *Pfr* state then reverts back into relaxed *Pr* state either by thermal dark reversion or by irradiation with far-red light [65]. Several engineered canonical BphPs are unable to convert to *Pfr* state and display only *Pr* state. The bathy BphPs adopt stable *Pfr* state in dark and yield *Pr* state upon irradiation with far-red light. The *Pr* state then converts back into relaxed *Pfr* state either by thermal dark reversion or by irradiation with red light [20]. In engineered bathy BphPs, like PAiRFP1 and PAiRFP2, the *Pr* and *Pfr* states coexist in the resting state.

**Figure 9:**
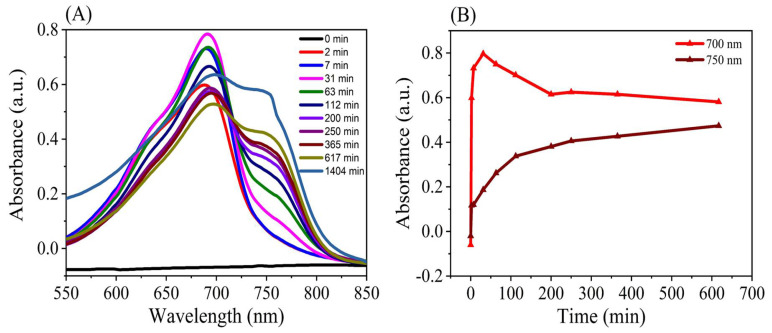
(**A**) Assembly of biliverdin (BV) with apoprotein of PAiRFP1 and (**B**) dynamic curve of the formation of both *Pr* at 700 nm and *Pfr* at 750 nm.

Usually, these two states cannot coexist in dark because they exhibit different thermodynamic stability. Activation barrier occurs (Ea) between *Pr* and *Pfr* on the ground state. However, once BphP is activated to the excited state, Ea will become smaller, therefore resulting in light-induced isomerization from *Pr* to *Pfr* on the excited state. That is called “photoconversion”. After *Pfr* is formed, it can revert back to *Pr* via light-dependent or light-independent way that is called dark recovery. The fact that dark recovery can happen infers that the activation energy Ea′ is not too high. The contrary situation was observed for Bathy BphPs. The energy of both Ea and Ea′ changes with BphP species and with the microenvironment surrounding BV. Dark recovery is related to dynamics or kinetics, where the rate is dependent on the activation energy between *Pr* and the intermediates. Therefore, we cannot exclude the possibility that *Pr* and *Pfr* states can coexist in resting state in PAiRFPs. Dark recovery also depends on temperature in canonical or pH- in bathy BphPs [66,67]. We have also studied the assembly dynamics of BV with apoprotein of PAiRFP1. As shown in Figure 9A,B, *Pr* state was formed quickly and then converted to *Pfr* state.

Almost all BphPs experience two reversible photocycles such as *Pr*→*Pfr* and *Pfr*→*Pr*. Low temperature X-ray crystallographic data, Fourier-transform infrared spectroscopy (FTIR), and Raman spectroscopy results showed that *Pfr* can be phototransformed into *Pr* mainly via three intermediates including Lumi-F, Meta-Fa, and Meta-Fb. In addition, *Pr* experiences Lumi-R, Meta-R, and Meta-Rc to reach *Pfr* state. Piatkevich et al. used 77K and 245K absorption spectroscopic methods to investigate the two photocycles in both PAiRFP1 and PAiRFP2. It was found that *Pfr* could switch to *Pr* smoothly. Nevertheless, the *Pr*→*Pfr* photoconversion was blocked at the Meta-Ra intermediate in PAiRFP1. Since Meta-Ra→Meta-Rc was the rate-determining step for the formation of *Pfr*, the *Pr*→*Pfr* photoconversion failed in PAiRFP1. Instead, Meta-Ra returned back to the initial *Pr* state. This explained why *Pr* state of PAiRFP1 could still emit NIR fluorescence even when the PHY domain was present. In spite of that, it was unclear about the key factors that blocked Meta-Ra→Meta-Rc→*Pfr* conversion in PAiRFP1. Some experimental methods such as femtosecond transient absorption spectroscopy, femtosecond Raman spectroscopy, and nanosecond flash photolysis are required to gain deeper understanding on the time scale of *fs*, *ps*, and *ns* to unravel this mechanism. Additionally, some theoretical calculations will also provide useful information regarding the structure and stability of both Meta-Ra and Meta-Rc.

**Figure 10:**
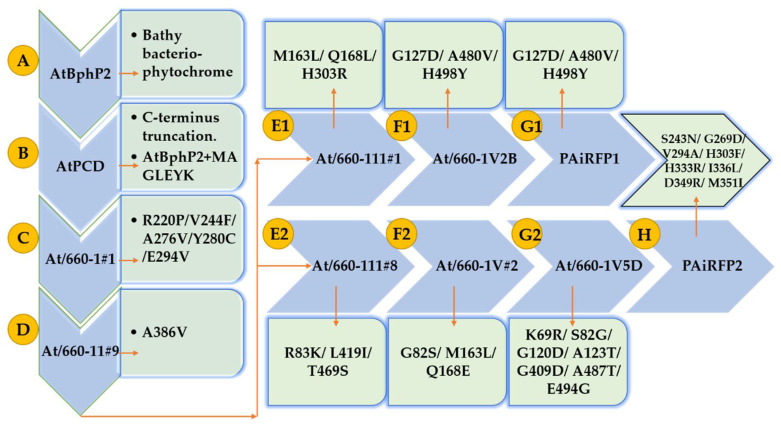
Molecular directed evolution of PAiRFP1 and PAiRFP2 presenting amino acid substitutions introduced during each round from (**A**) to (**H**) [17].

Our present study suggested that after several rounds of mutations by Piatkevich et al., the mutants Q168L, V244F, and A480V will enhance the molecular stability and flexibility of Agp2. A series of each round of library of different mutants that are constructed by random mutagenesis are explained in Figure 10. We also found that structures of PAiRFP1 and PAiRFP2 are highly deviated than Agp2 from its native conformation, but PAiRFP1 exhibited more tight packing as compared to Agp2 and PAiRFP2 during 100 ns MD simulations. The hydrogen bonds between main chain and side chains of PAiRFP1 were less than Agp2 and PAiRFP2, which led to the exposure of the hydrophobic region of PAiRFP1 core to more solvent molecules. Furthermore, the eigenvalues and trace of covariance matrix were found higher for PAiRFP1 and PAiRFP2 as compared to Agp2. It means that different point mutations increase the random atomic fluctuations of these PA NIR FPs during their directed molecular evolution from Agp2. The detailed computational analysis provided useful understanding structure dynamics of Agp2, PAiRFP1, and PAiRFP2. We found that several mutations are stabilizing, destabilizing, favorable, and unfavorable. The predicted mutations such as Q168L, V244F, and A480V in Agp2 will enhance the molecular stability and flexibility of protein. It is possible to further design a better PA NIR FPs from Agp2 than PAiRFP1 and PAiRFP2.

We carried out structural analysis and found that the average SASA values with respect to backbone for Agp2, PAiRFP1, and PAiRFP2 were found to be 248.60, 252.88, and 250.42 nm^2^, respectively. PAiRFP1 and PAiRFP2 had higher SASA than Agp2. Similar phenomenon was observed for the free energy of solvation. The hydrogen bond and secondary structural analysis revealed that the volume of PAiRFP1 and PAiRFP2 are slightly larger than Agp2. Principal component analysis showed that both PAiRFP1 and PAiRFP2 exhibit higher eigenvalues and the trace of covariance matrix. It suggested that there are more random atomic fluctuations in PAiRFP1 and PAiRFP2. All of these aspects might be the reason why PAiRFP1 and PAiRFP2 have slower dark recovery rate than Agp2. The dark recovery is related to dynamics or kinetics, where the rate is dependent on the activation energy between *Pr* and the intermediates. Further study is needed to unravel this phenomenon. 

Although we have performed detailed comparative analysis of Agp2, PAiRFP1, and PAiRFP2, there are many possible research limitations. The experimental validations to support this computational prediction are further required. The results of computational analysis can have several variations when it is executed by different software using different force fields. Additionally, the theoretical prediction may have several variations when it comes to experimental level such as stabilizing and destabilizing effects of amino acids in the structures of Agp2, PAiRFP1, and PAiRFP2. The difference may arise due to different positions of amino acids in the structure of protein.

## 5. Conclusions

In this study, a comprehensive molecular exploration and comparative studies of Agp2, PAiRFP1, and PAiRFP2 are carried out. The PAiRFP1 was expressed and purified, and the fluorescence intensity and time-dependent dynamic curve for PAiRFP1 was obtained. It was found that both PAiRFP1 and PAiRFP2 perform photoactivation behavior because *Pr* and *Pfr* states are coexisting in resting state, and *Pfr* state can be photoconverted into *Pr* state, which emits fluorescence upon absorption of far-red light. The computational findings suggested that the structure of PAiRFP1 and PAiRFP2 are more deviated and the hydrophobic cores that were buried in PAiRFP1 and PAiRFP2 were exposed to solvent molecules during the MD simulations. We found that some mutations at residues Q168, V244, and A480 of Agp2 are more useful as it enhances the molecular stability and flexibility. This study is helpful to gain understanding of structural changes during the evolution of these photoactivatable of NIR FPs.

## Figures and Tables

**Table 1:** Analysis of the mutational sites of PAiRFP1 and PAiRFP2 and their comparison with wild-type Agp2 in “period clock protein” (Per), “aromatic hydrocarbon receptor nuclear *trans*locator” (ARNT), and “single-minded protein” (Sim) (PAS) domain.

S. No.	Domains	Agp2			PAiRFP1			PAiRFP2		
		Sites	Hydrogen Bonds	Van der Waals Interactions	Mutation Sites	H-Bonds	Van der Waals Interactions	Mutation Sites	H-Bonds	Van der Waals Interactions
1	PAS domain	K69	H72 D73	G67 K68 L70 V71	–	–	–	R69	H72 D73	G67 K68 L70 V71
2		R83	–	T81 G82 T84 T85	–	–	–	K83	–	T81 G82 T84 R86
3		G120	–	S119 D122 S121	–	–	–	D120	S121	S119 D122 S286

**Table 2:** Analysis of the mutational sites of PAiRFP1 and PAiRFP2 and their comparison with wild-type Agp2 in cGMP phosphodiesterase/adenyl cyclase/FhlA cGMP specific phosphodiesterase/adenylate cyclase and (FhIA) formate hydrogen lyase *trans*cription activator (GAF) domain.

S. No.	Domains	Agp2			PAiRFP1			PAiRFP2		
		Sites	Hydrogen Bonds	Van der Waals Interactions	Mutation Sites	H-Bonds	Van der Waals Interactions	Mutation Sites	H-Bonds	Van der Waals Interactions
1	GAF domain	A123	–	D122 Q124 P125 P288	–	–	–	T123	–	D122 Q124 P125 P288
2		G127	R130 K131	Q124 P125 L126 T128 A129	D127	R130 K131	Q124 P125 L126 T128 A129	–	–	–
3		S 141	S144 L145	A139 D140 V142 E143 L307	R141	S144 L145	A139 D140 V142 E143 L307	–	–	–
4		M163	Y165 A276	T150 V162 I164 S177 E178 F187 L274 I275	L163	V276	T150 V162 I164 S177 E178 F187 L274 I275	L163	V276	T150 V162 I164 S177 E178 F187 I275
5		Q168	R166 G171 A172	F167 E169 D170 G173 K174 Y191	L168	G171 A172	F167 E169 D170 G173 K174 Q475 W466 R166	E168	R166 G171 A172	F167 E169 D170 G173 K174 Y191 Q475
6		A203	Q199 Q200 L206 K207	A201 R202 L204 Y205	V203	Q199 Q200 L206 K207	A201 R202 L204 Y205	–	–	–
7		G218	D215	A216 S217 T219 R220	S218	D215	A216 S217 T219 P26	–	–	–
8		R220	–	I24 P26 G218 T219 I221 S243 V244 P246 R253	P220	–	I24 P26 T219 I221 S243 P222	P220	–	I24 P26 T219 I221 S243 Y23
9		S 243	R211	P21 I24 Y23 Q25 L241 R242	–	–	–	N243	Y23 R211 F244	P21 I24 Q25 L236 L241 R242
10		V244	–	R211 R242 S243 S245 P246 H248	F244	–	R211 S243 S245 P246 H248 C249	F244	–	Q25 I212 R242 S245 P246 H248 C249
11		G269	–	L206 I266 V267 A270	–	–	–	D269	–	L206 I266 V267 A270
12		A276	–	R161 V162 I164 M261 S262 L274 I275 C277	V276	–	R161 V162 I164 M261 S262 L274 I275 C277	V276	–	R161 V162 I164 M261 S262 I263 L274 I275
13		Y280	D160 R161	E185 G256 A258 H279 S281	C280	D160	L184 G256 V257 A258 H279 S281	C280	D160	L184 G256 V257 A258 H279 S281
14		E294	R290 I291 G297 E298	T209 L210 A292 A293 M295 F296	V294	R290 I291 G297 E298	T84 T85 T209 A292 A293 M295 F296	A294	R290 I291 G297 E298	T209 L210 A292 A293 M295 F296
15		H303	F299 F300 V306 L307	I136 S301 M302 L304 Q305	R303	F299 F300 V306 L307	I136 A139 S301 M302 L304 Q305	F303	F299 F300 V306 L307	R137 S301 M302 L304 Q305

**Table 3:** Analysis of the mutational sites of PAiRFP1 and PAiRFP2 and their comparison with wild-type Agp2 in phytochrome-specific (PHY) domain.

S. No.	Domains	Agp2			PAiRFP1			PAiRFP2		
		Sites	Hydrogen Bonds	Van der Waals Interactions	Mutation Sites	H-Bonds	Van der Waals Interactions	Mutation Sites	H-Bonds	Van der Waals Interactions
1	PHY domain	H333	A330	A331 H332 A334 N335 I336 H499	–	–	–	R333	A330	L329 A331 H332 A334 N335 L339 D342 H499
2		I336	L339 L340	A334 N335 E337 E338 V495	–	–	–	L336	L339 L340	A334 N335 E337 E338 A496
3		D349	D346	H318 F347 A348 L350 M351	–	–	–	R349	D346	F347 A348 L350 I351
4		M351	–	A348 P352 C353 L415 D481 A485	–	–	–	I 351	–	A348 L350 P352 C353 R431 W477 D481
5		A386	F382 V383	A384 S385 S387 E388	V386	F382 V383	A384 S385 S387 E388	V386	F382 V383	A384 S385 S387 E388
6		G409	–	Y407 A408 T410 A411 K432	–	–	–	D409	–	Y407 A408 T410 A411 K432 E433 Q436
7		L419	D425	L,340 L358 V360 I417 S420 Y426 L427 L492	–	–	–	I419	D425	I417 P418 S420 Y426 L427 L492
8		T469	I465 W466	P193 K467 E468 V470 R471 Q473	–	–	–	S469	I465 W466	K467 E468 V470 R471 Q473
9		A480	E483 I484	S478 E479 D481 R482	V480	E483 I484	I316 S478 E479 D481 R482	–	–	–
10		A487	E483 I484 I490 A491	L323 A485 E486 A488 R489	–	–	–	T487	E483 I484 A491	A319 H320 L323 A485 E486 A488 R489 I490
11		E494	I490 A491 F497	L327 L492 V493 V495 A496 H499	–	–	–	G494	I490 A491 F497 H498.	L327 L492 V493 V495 A496 H498
12		H498	E494 V495 E501 H502	L327 A496 F397 H499 S500	Y498	E494 Y508	V495 A496 H499, S500	–	–	–

**Table 4:** Prediction of molecular flexibility and stability of the mutational sites of PAiRFP1 and their comparison with wild-type Agp2.

S. NO.	Agp2	PAiRFP1	ΔΔG ENCoM (kcal/mol)	ΔΔS ENCoM (kcal.mol^−1^.K^−1^)	Molecular Flexibility	ΔΔG DynaMut (kcal/mol)	Outcome	DeepDDG (kcal/mol)	Outcome
1	G127	G127D	0.04	−0.05	Decrease	0.021	Stabilizing	0.220	Stable
2	S141	S141R	−0.073	0.092	Increase	−0.096	Destabilizing	−0.347	Unstable
3	M163	M163L	−0.158	0.197	Increase	−0.391	Destabilizing	−1.061	Unstable
4	Q168	Q168L	0.138	−0.172	Decrease	1.204	Stabilizing	0.298	Stable
5	A203	A203V	0.128	−0.16	Decrease	0.308	Stabilizing	−0.274	Unstable
6	G218	G218S	0.209	−0.261	Decrease	−0.244	Destabilizing	−0.661	Unstable
7	R220	R220P	−0.741	0.926	Increase	−0.179	Destabilizing	0.361	Stable
8	V244	V244F	0.519	−0.649	Decrease	1.154	Stabilizing	−1.383	Unstable
9	A276	A276V	0.184	−0.231	Decrease	0.791	Stabilizing	−0.495	Unstable
10	Y280	Y280C	−1.03	1.287	Increase	−0.188	Destabilizing	−0.786	Unstable
11	E294	E294V	−0.371	0.464	Increase	−0.032	Destabilizing	−0.614	Unstable
12	H303	H303R	−0.22	0.275	Increase	−0.955	Destabilizing	−0.072	Unstable
13	A386	A386V	0.127	−0.159	Decrease	0.09	Stabilizing	0.154	Stable
14	A480	A480V	0.373	−0.466	Decrease	0.964	Stabilizing	0.147	Stable
15	H498	H498Y	0.085	−0.106	Decrease	0.994	Stabilizing	−1.230	Unstable

**Table 5:** Prediction of molecular flexibility and stability of the mutational sites of PAiRFP2 and their comparison with wild-type Agp2.

S. NO.	Agp2	PAiRFP2	ΔΔG ENCoM (kcal/mol)	ΔΔS ENCoM (kcal.mol^−1^.K^−1^)	Molecular Flexibility	ΔΔG DynaMut (kcal/mol)	Outcome	DeepDDG (kcal/mol)	Outcome
1	K69	K69R	−0.263	0.328	Increase	−0.445	Destabilizing	−0.148	Stable
2	R83	R83K	−0.077	0.097	Increase	−0.948	Destabilizing	−0.044	Unstable
3	G120	G120D	0.078	−0.098	Decrease	0.671	Stabilizing	0.043	Stable
4	A123	A123T	−0.034	0.042	Increase	0.022	Stabilizing	−0.050	Unstable
5	M163	M163L	−0.158	0.197	Increase	−0.391	Destabilizing	−1.061	Unstable
6	Q168	Q168E	0.104	−0.13	Decrease	0.097	Stabilizing	−0.492	Unstable
7	R220	R220P	−0.741	0.926	Increase	−0.179	Destabilizing	0.361	Stable
8	S243	S243N	0.073	−0.092	Decrease	0.578	Stabilizing	−2.434	Unstable
9	V244	V244F	0.519	−0.649	Decrease	1.154	Stabilizing	−1.383	Unstable
10	G269	G269D	−0.173	0.217	Increase	−1.562	Destabilizing	−1.040	Unstable
11	A276	A276V	0.184	−0.231	Decrease	0.791	Stabilizing	−0.495	Unstable
12	Y280	Y280C	−1.03	1.287	Increase	−0.188	Destabilizing	−0.786	Unstable
13	E294	E294A	−0.573	0.717	Increase	−0.854	Destabilizing	−0.667	Unstable
14	H303	H303F	0.155	−0.193	Decrease	0.479	Stabilizing	0.804	Stable
15	H333	H333R	−0.138	0.172	Increase	−0.254	Destabilizing	−0.402	Unstable
16	I336	I336L	0.203	−0.254	Decrease	1.153	Stabilizing	0.460	Stable
17	D349	D349R	0.138	−0.172	Decrease	0.824	Stabilizing	−0.044	Unstable
18	M351	M351I	−0.369	0.461	Increase	−0.516	Destabilizing	0.666	Stable
19	A386	A386V	0.127	−0.159	Decrease	0.09	Stabilizing	0.154	Stable
20	G409	G409D	0.229	−0.286	Decrease	0.803	Stabilizing	0.608	Stable
21	L419	L419I	0.016	−0.019	Decrease	0.173	Stabilizing	−0.087	Unstable
22	T469	T469S	−0.141	0.177	Increase	−0.67	Destabilizing	−0.487	Unstable
23	A487	A487T	0.33	−0.412	Decrease	0.627	Stabilizing	−0.032	Unstable
24	E494	E494G	−0.313	0.391	Increase	−1.362	Destabilizing	−1.329	Unstable

**Table 6:** The electrostatic, van der Waals, and covalent residual interactions of BV in *cis* and *trans* form with Agp2, PAiRFP1, and PAiRFP2, respectively.

Electrostatic Interactions		Van der Waals Interactions		Covalent Bonds	
**Agp2-BV*cis***	**Agp2-BV*trans***	**Agp2-BV*cis***	**Agp2-BV*trans***	**Agp2-BV*cis***	**Agp2-BV*trans***
Y165 S195 N196 R211 H248 S245 H278 R456	E16 Y165 D196 Y205 R211 H248 H278 R456	C13 D14 F187 Q190 F192 I197 P198 Y205 I213 V244 A276 P459 S462	Y191 F192 S195 I197 P198 F244 S245 I247 Y251 V276	C13	C13
**PAiRFP1-BV*cis***	**PAiRFP1-BV*trans***	**PAiRFP1-BV*cis***	**PAiRFP1-BV*trans***	**PAiRFP1-BV*cis***	**PAiRFP1-BV*trans***
S195 D196 R211 H248 M255 H278 S260 R456	Y165 S195 D196 R211 S245 H248 H278 R456	C13 D14 I18 F192 I197 P198 A201 Y205 F244 I247 Y251 V276 P459 S462	C13 D14 Q190 F192 I197 P198 Y205 I213 V244 A276 S462	C13	C13
**PAiRFP2-BV*cis***	**PAiRFP2-BV*trans***	**PAiRFP2-BV*cis***	**PAiRFP2-BV*trans***	**PAiRFP2-BV*cis***	**PAiRFP2-BV*trans***
E16 Y165 Q190 D196 Y205 R211 H248 Y251 H278 R456 S462	S195 D196 R211 H248 Y251 S260 H278 R456 S462	F187 F192 S195 I197 P198 F244 S245 I247 M255 V276 P459	C13 D14 I18 F192 I197 P198 A201 Y205 F244 I247 V276 P459	C13	C13

**Table 7:** Percentage of residues in Agp2, PAiRFP1, and PAiRFP2 participated in average structure formation during 100 ns molecular dynamics (MD) simulations.

Percentage of Secondary Structure (SS %)								
Protein	Structure *	Coil	β-Sheet	β-Bridge	Bend	Turn	α-Helix	3_10_-Helix
Agp2	65	21	21	1	13	9	34	1
PAiRFP1	65	20	21	2	13	10	32	1
PAiRFP2	65	21	20	2	13	9	34	1

* Structure = α-helix + β-sheet + β-bridge + turn.

## Supplementary Materials

Supplementary Materials can be found at https://www.mdpi.com/2218-273X/10/9/1286/s1, Figure S1: The 3D structure of (A) Agp2, (B) PAiRFP1, and (C) PAiRFP2 predicted by homology modelling. Ramachandran plot of (D) Agp2, (E) PAiRFP1, and (F) PAiRFP2 indicated that they have 93.5% of entire residues are in most favored regions.

## Abbreviations

MD	Molecular dynamics
BphPs	Bacteriophytochrome photoreceptors
NIR	Near infrared
RMSD	Root mean square deviation
