# Supplementary material for: Comparative Analysis of Bacteriophytochrome Agp2 and Its Engineered Photoactivatable NIR Fluorescent Proteins PAiRFP1 and PAiRFP2
Source: Biomolecules. 2020 Sep 7;10(9):1286. doi: 10.3390/biom10091286 (PMC7564321; doi:10.3390/biom10091286)
Supplement: Supplementary file 1 [file biomolecules-10-01286-s001.pdf]

biomolecules

MDPI

Article

Comparative Analysis of Bacteriophytochrome Agp2 and Its Engineered Photoactivatable NIR Fluorescent Proteins PAiRFP1 and PAiRFP2

Faez Iqbal Khan [1,†], Fakhrul Hassan [2,†], Razique Anwer [3], Feng Juan [2] and Dakun Lai [1,*]

1 School of Electronic Science and Engineering, University of Electronic Science and Technology of China, Chengdu 610054, Sichuan, China; khanfaeziqbal@gmail.com

2 School of Life Science and Technology, University of Electronic Science and Technology of China, Chengdu 610054, Sichuan, China; fakhar_ibneadam@yahoo.com (F.H.); fengjuan@uestc.edu.cn (F.J.)

3 Department of Pathology, College of Medicine, Imam Mohammad ibn Saud Islamic University (IMSIU), Riyadh 13317, Saudi Arabia; razainuddin@imamu.edu.sa

* Correspondence: dklai@uestc.edu.cn; Tel.: +86-182-0052-9516

† Contributed equally.

Received: date; Accepted: date; Published: date

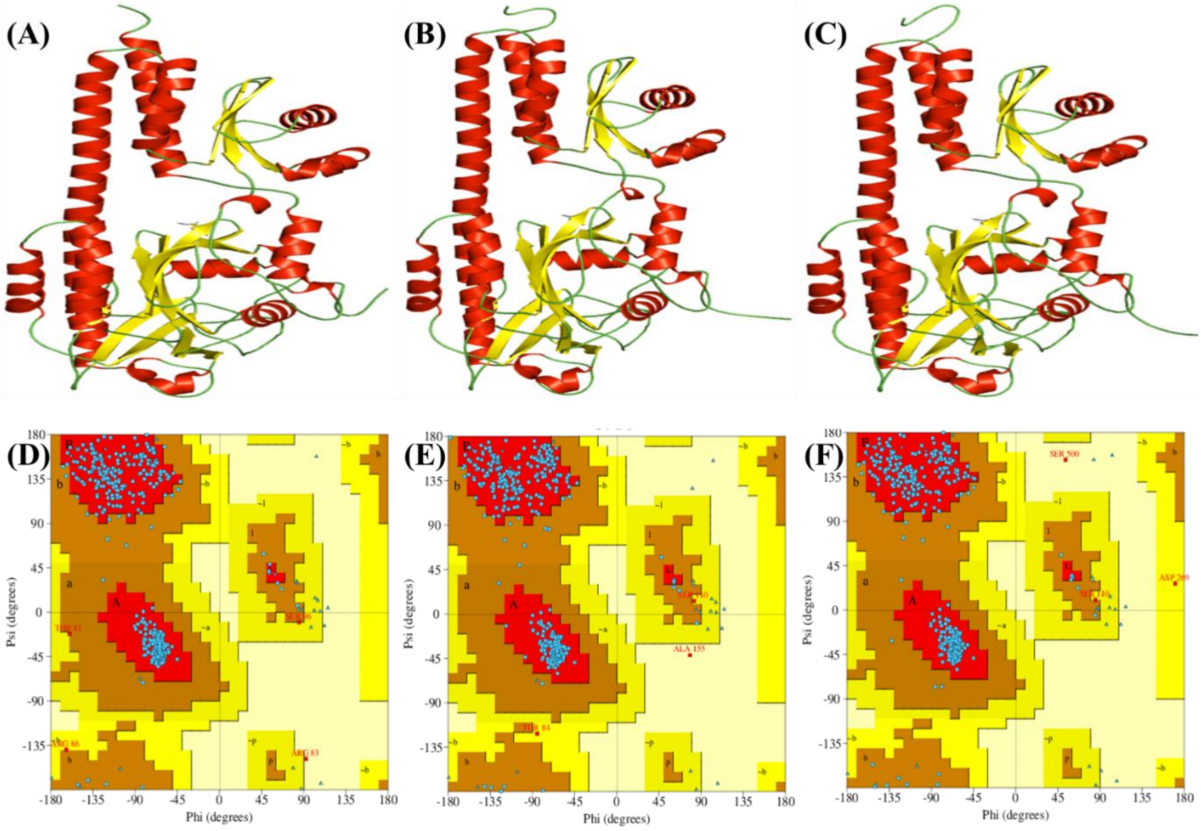

Figure S1. The 3D structure of (A) Agp2, (B) PAiRFP1, and (C) PAiRFP2 predicted by homology modelling. Ramachandran plot of (D) Agp2, (E) PAiRFP1, and (F) PAiRFP2 indicated that they have 93.5% of entire residues are in most favored regions.
